# DNA Damage Sensor **γ**-H2AX Is Increased in Preneoplastic Lesions of Hepatocellular Carcinoma

**DOI:** 10.1155/2013/597095

**Published:** 2013-03-03

**Authors:** Yasunobu Matsuda, Toshifumi Wakai, Masayuki Kubota, Mami Osawa, Masaaki Takamura, Satoshi Yamagiwa, Yutaka Aoyagi, Ayumi Sanpei, Shun Fujimaki

**Affiliations:** ^1^Department of Medical Technology, Niigata University Graduate School of Health Sciences, 2-746 Asahimachi-dori, Niigata 951-8518, Japan; ^2^Division of Digestive and General Surgery, Niigata University Graduate School of Medical and Dental Sciences, 2-746 Asahimachi-dori, Niigata 951-8518, Japan; ^3^Division of Pediatric Surgery, Niigata University Graduate School of Medical and Dental Sciences, 2-746 Asahimachi-dori, Niigata 951-8518, Japan; ^4^Division of Gastroenterology and Hepatology, Niigata University Graduate School of Medical and Dental Sciences, 2-746 Asahimachi-dori, Niigata 951-8518, Japan

## Abstract

*Background*. Phosphorylated histone H2AX (**γ**-H2AX) is a potential regulator of DNA repair and is a useful tool for detecting DNA damage. To evaluate the clinical usefulness of **γ**-H2AX in hepatocellular carcinoma (HCC), we measured the level of **γ**-H2AX in HCC, dysplastic nodule, and nontumorous liver diseases. *Methods*. The level of **γ**-H2AX was measured by immunohistochemistry in fifty-eight HCC, 18 chronic hepatitis, 22 liver cirrhosis, and 19 dysplastic nodules. Appropriate cases were also examined by fluorescence analysis and western blotting. *Results*. All cases with chronic liver disease showed increased levels of **γ**-H2AX expression. In 40 (69.9%) of 58 cases with HCC, the labeling index (LI) of **γ**-H2AX was above 50% and was inversely correlated with the histological grade. Mean **γ**-H2AX LI was the highest in dysplastic nodule (74.1 ± 22.1%), which was significantly higher than HCC (*P* < 0.005). Moreover, **γ**-H2AX was significantly increased in nontumorous tissues of HCC as compared with liver cirrhosis without HCC (62.5 ± 24.7%, from 5.1 to 96.0%, *P* < 0.005). *Conclusions*. **γ**-H2AX was increased in the preneoplastic lesions of HCC and might be a useful biomarker for predicting the risk of HCC.

## 1. Introduction

Hepatocellular carcinoma (HCC) is one of the most common malignancies in developing and industrial countries and is increasing worldwide [1–4]. HCC is unique as it frequently reoccurs after treatment irrespective of the different etiological factors including hepatitis virus B (HBV) and C (HCV), alcohol abuse, and nonalcoholic steatohepatitis [2, 4, 5]. One possible reason for the frequent recurrence of HCC might be due to many patients being affected with hepatitis virus-associated chronic liver inflammation [1–3].

To date, many reports have described a possible relationship between hepatitis virus and DNA damage. For example, HBV has been reported to directly regulate the DNA damage response in host cells [6]. HBV stimulates ATM- and Rad3-related protein kinase (ATR)andcheckpoint kinase 1 (Chk1) pathways [7], leading to the acquisition of strengthened survival against DNA damage. Moreover, HBV X gene product (HBX), widely recognized as a possible viral carcinogen [8, 9], plays a critical role in the phosphorylation and inactivation of Rb via activating p38 mitogen-activated protein kinase [10]. HBX also binds and inhibits the functional efficiency of p53 [11, 12], leading to DNA damage accumulation in HBV-infected cells. HCV has been also reported to be involved in the deregulation of the DNA repair system. HCV nonstructural proteins, NS3 and NS4A, inhibit Ataxia-telangiectasia-mutated (ATM) kinase in response to DNA damage [13]. HCV core protein inhibits the functional formation of the Mre11/NBS1/Rad50 complex, which causes the ATM-mediated DNA repair system to be markedly impaired in HCV-infected cells [14]. Together, these lines of evidence strongly suggest that DNA damage response machinery is significantly involved in hepatocarcinogenesis and might be used as biomarkers for predicting the risk of HCC development.

Recently, several studies reported that the level of oxidative DNA damage is a good biomarker. For example, hepatic 8-oxo-2′-deoxyguanosine (8-OHdG), an oxidized derivative of deoxyguanosine, which reflects oxidative stress, was closely correlated with the risk of HCC recurrence after surgery [15, 16]. To search for more sensitive and reliable biomarkers of DNA damage, we investigated the levels of *γ*-H2AX in HCC tissues, which mark the site of DNA double-strand breaks and evoke the DNA repair system [17, 18]. To address whether *γ*-H2AX might be a good indicator for the risk of HCC development, we also examined and compared the level of *γ*-H2AX in nontumorous chronic liver diseases.

## 2. Materials and Methods

### 2.1. Patients

The pathological diagnoses and analyses of dysplastic nodule and HCC were made according to the General Rules for the Clinical and Pathological Study of Primary Liver Cancer [19]. HCC tissue samples were obtained from 58 patients (7 cases with hepatitis B virus-positive, 35 with hepatitis C virus-positive, and 16 with unknown etiology; 9 females, 49 males; mean age, 62 ± 9) that underwent hepatic resection at Niigata University Medical and Dental Hospital. Tissue samples of dysplastic nodules were obtained from 19 patients (2 cases with hepatitis B virus-positive, 12 with hepatitis C virus-positive, and 5 with unknown etiology; 13 males and 6 females; mean age, 63 ± 8) by ultrasound-guided biopsy (Table 1). Tissue samples of 18 cases with chronic hepatitis (6 with hepatitis B virus-positive and 10 with hepatitis C virus-positive; 14 males and 4 females) and 22 with liver cirrhosis (4 with hepatitis B virus-positive and 18 with hepatitis C virus-positive; 19 males and 3 females) were obtained by ultrasound-guided or laparoscopic biopsy. All tissue samples were fixed in formalin, and the tissue sections were subjected to hematoxylin and eosin staining for histopathological evaluation by two pathologists. Freshly frozen tissues were obtained from 12 cases with HCC and 8 with liver cirrhosis and were used for western blot analysis. Normal liver tissue samples were surgically obtained from 5 individuals without liver disease. Informed consent was obtained from all the human subjects included in the study under an Institutional Review Board-approved protocol, and the study protocol conformed to the ethical guidelines of the 1975 Declaration of Helsinki as reflected in *a priori* approval by the institution's human research committee.

### 2.2. Immunohistochemical Analysis

Tissue sections were deparaffinized in xylene, rehydrated in alcohol, and quenched in 3% hydrogen peroxide with methanol to block endogenous peroxidase activity. Slides were heated in a microwave in 10 mm sodium citrate (pH 6.5) for antigen retrieval. Immunohistochemical reactions were performed by immersing tissue sections in 5% normal goat serum for 60 minutes and incubating them at 4°C overnight with mouse anti-phospho-histone H2AX monoclonal antibody (Ser139) clone JBW301 (Upstate Biotech, Charlottesville, VA) at a dilution of 1 : 500 in blocking buffer. As a negative control, control mouse immunoglobulin G (Dako Cytomation, Glostrup, Denmark) was used instead of the primary antibody. After the sections were rinsed, a secondary antibody from the Vectastain Elite ABC Kit (Vector Laboratories, Burlingame, CA) was applied, and color development was performed using 3,3-diaminobenzidine (Sigma Chemical Co., St Louis, MO). Counterstain was provided by staining with hematoxylin. Labeling indices (LIs) for *γ*-H2AX were determined as the number of positive nuclei in 100 hepatocytes or tumor cells in 3 randomly selected fields. In HCC cases, the patients were divided into two groups according to the levels of *γ*-H2AX LI, as low expressors (LI  <50%) and high expressors (LI >50%).

### 2.3. Immunofluorescence Staining

For immunofluorescence analysis, appropriate tissue slides were incubated in 100 mm glycine for 15 minutes, three times to reduce fluorescent background. Slides were reacted with the same primary antibody as used for immunohistochemistry and washed in tris-buffered saline containing 0.05% Tween-20 3 times for 5 minutes to reduce background. They were incubated for 30 minutes with Alexa Fluor 488 goat anti-mouse IgG (H + L) (Molecular Probes, Eugene, OR) in the dark and mounted with 0.2 *μ*g/mL 4′-6-diamidino-2-phenylindole (DAPI). Immunofluorescence images were visualized by fluorescence microscope (BZ-9000; Keyence, Osaka, Japan).

### 2.4. Western Blotting

Liver tissues were homogenized using a TissueRuptor (Qiagen, Valencia, CA, USA) with a buffer containing 20 mm Tris-HCl (pH 7.4), 150 mm NaCl, 2 mm EGTA, 5 mm *β*-glycerophosphate, 1 mm MgCl_2_, 1% Triton X-100, 1 mm sodium orthovanadate, 10 *μ*g/mL protease inhibitors, 1 *μ*g/mL aprotinin, 1 *μ*g/mL leupeptin, and 1 *μ*g/mL pepstatin. Lysates were cleared by centrifugation, and the supernatants containing 20 *μ*g of protein were electrophoresed on 5–20% SDS-polyacrylamide gels. After samples were blotted onto Hybond-P membranes (GE Healthcare, Milwaukee, WI), membranes were incubated with rabbit anti-*γ*-H2AX polyclonal antibody (Bethyl Lab. Inc., Montgomery, TX). Protein blots were visualized using an enhanced ECL Western blotting detection system (GE Healthcare), and equal amounts of the protein loading were confirmed by reprobing with anti-*β*-actin antibody (Sigma Chemical Co.).

### 2.5. Statistical Analysis

Data were analyzed using SPSS software (Statistical Product and Service Solutions 11.5 for Windows; SPSS Inc., Chicago, IL). Chi-square test was used for examining the association between the status of *γ*-H2AX and clinic-pathologic features in HCC. When appropriate, a Mann-Whitney *U*-test or independent Student's *t*-test was used to test for statistical differences between the groups.

## 3. Results

### 3.1. *γ*-H2AX Expression Is Increased in HCC

**Figure 1:**
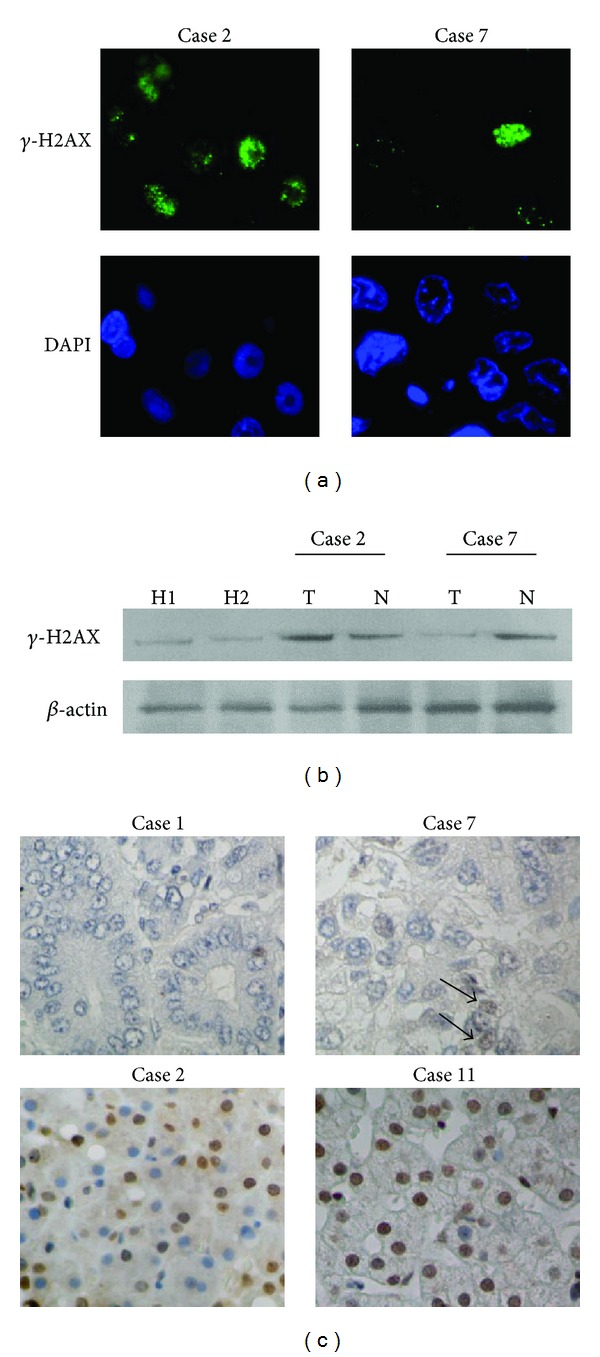
*γ*-H2AX is expressed at different levels in HCC. (a) Immunofluorescence staining shows that phosphorylated histone H2AX (*γ*-H2AX) is located in the nuclei of HCC cells (green) (original magnification ×100). Case  2: HCC with increased *γ*-H2AX expression; Case 7: HCC with sparse expression of *γ*-H2AX. DAPI (blue): nucleus counterstain. (b) Representative data of western blotting for *γ*-H2AX in liver tissues. H1 and H2: healthy livers; Cases  2 and 7: HCC cases. T: tumor tissues; N: adjacent nontumorous liver tissues. (c) Immunohistochemical staining of *γ*-H2AX. Cases  1 and 7: HCC cases with negative to low expression of *γ*-H2AX. Cases  2 and 11: HCCs with high expression of *γ*-H2AX (original magnification ×40). Arrows indicate positive staining in the nuclei.

Immunofluorescence analysis demonstrated that *γ*-H2AX appeared as diffuse and discrete foci in the nuclei of HCC cells (Figure 1(a)). The results of western blotting correlated with the immunofluorescence staining (Figure 1(b)), confirming the potential reliability for detecting *γ*-H2AX in the HCC samples. Immunohistochemical analysis showed that the mean value of LI for *γ*-H2AX in HCC was 56.2 ± 31.4% (range from 3.0 to 95.1%), which was significantly increased compared with normal livers, LI 1.0 ± 0.6%. Fifty-eight HCC patients were categorized into two groups, 40 (69.0%) characterized by significantly increased levels of *γ*-H2AX expression (high expressors; LI >50%), and 18 (31.9%) with very low to negative expression (low expressors; LI <50%) (Figure 1(c)). *γ*-H2AX expression levels showed no positive correlation with clinical features in HCC patients (Table 2); however, there was an inverse relationship between the histological grade of the tumors (*P* = 0.011) (Table 2).

### 3.2. *γ*-H2AX Is Increased in Chronic Liver Diseases and Dysplastic Nodules

**Figure 2:**
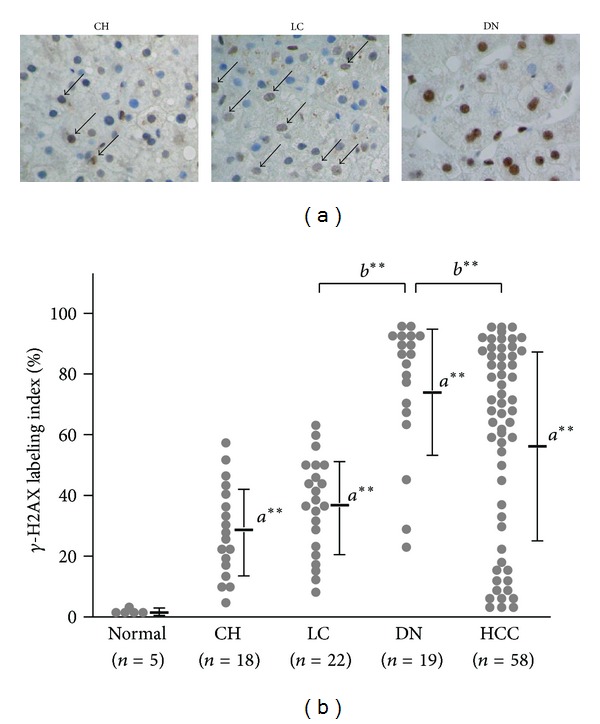
*γ*-H2AX is significantly increased in preneoplastic lesions in the liver. (a) Representative images of immunostaining for *γ*-H2AX in non-HCC tissues. CH: chronic hepatitis; LC: liver cirrhosis; DN: dysplastic nodule (original magnification ×40). (b) Dot plots showing the *γ*-H2AX labeling index in normal livers (*n* = 5), chronic hepatitis (CH; *n* = 18), liver cirrhosis (LC; *n* = 22), dysplastic nodule (DN; *n* = 19), and HCC (*n* = 58). Horizontal bars depict the mean value, and vertical bars indicate the standard deviation. a**: *P* value of <0.01 versus normal livers; b**: *P* value of <0.01 versus dysplastic nodule.

Immunohistochemical analysis showed that the LI of *γ*-H2AX in chronic hepatitis and liver cirrhosis was increased compared with normal livers (chronic hepatitis, 27.5 ± 15.8%, range from 5.0 to 59.3%, *P* < 0.005; liver cirrhosis, 56.2 ± 31.4%, range from 7.2 to 63.0%, *P* < 0.005, resp.). Intriguingly, dysplastic nodules showed a significantly increased *γ*-H2AX expression (74.1 ± 22.1%, range from 20.1 to 94.0%), which was significantly increased compared with those in liver cirrhosis (*P* < 0.005) and HCC (*P* < 0.005) (Figures 2(a) and 2(b)).

### 3.3. *γ*-H2AX Is Increased in Adjacent Nontumorous Liver Tissues from HCC Patients

**Figure 3:**
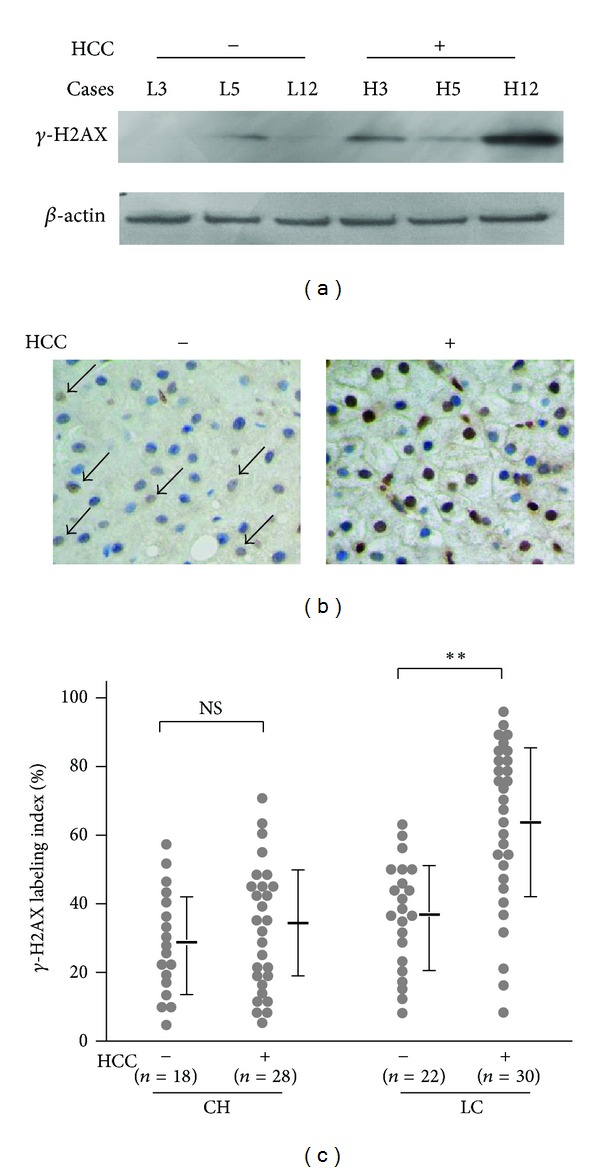
*γ*-H2AX is increased in the adjacent nontumorous liver tissues of HCC patients. (a) Representative data of western blotting for *γ*-H2AX. L3, 5, and 12: cases with liver cirrhosis without the coexistence of HCC; H3, 5, and 12: adjacent nontumorous liver tissues obtained from HCC patients. (b) Representative images of *γ*-H2AX immunostaining in liver tissues with and without the coexistence of HCC (original magnification ×40). (c) Dot plots showing the *γ*-H2AX labeling index. CH: chronic hepatitis without the coexistence of HCC (*n* = 18) and with HCC (*n* = 28); LC: liver cirrhosis without HCC (*n* = 22) and with HCC (*n* = 30). NS: not significant; **P < 0.01.

To investigate the clinical significance of *γ*-H2AX, we determined whether fundamental differences existed between the nontumorous liver tissues with and without the coexistence of HCC. Western blotting detected *γ*-H2AX in 3 (37.5%) of 8 liver cirrhosis patients with no evident HCC occurrence, while 9 (75%) of 12 HCC cases showed a detectable protein band for *γ*-H2AX in the adjacent nontumorous liver tissues (Figure 3(a)). Immunohistochemical analysis showed that the mean LI of *γ*-H2AX in nontumorous liver tissues obtained from HCC patients with chronic hepatitis was relatively but not statistically increased as compared with that obtained from individuals with chronic hepatitis without HCC occurrence (35.7 ± 17.2%, range from 8.0 to 65.5%, *P* = 0.11). Moreover, the mean LI of *γ*-H2AX in nontumorous tissues from HCC patients with liver cirrhosis was significantly increased when compared with the cases with liver cirrhosis without HCC (62.5 ± 24.7%, range from 5.1 to 96.0%, *P* < 0.005) (Figure 3(c)).

## 4. Discussion

Over the last few decades, alpha-fetoprotein (AFP), normally produced by the fetal liver and yolk sac in pregnant individuals, has commonly been used as a clinically available biomarker for HCC. Unfortunately, serum levels of AFP do not correlate well with the risk of the development of HCC, and reliable biomarkers have been long awaited for improving the poor prognosis of HCC. As DNA double-strand breaks accumulate during long periods of chronic inflammation, investigating the molecules involved in the DNA repair system for use as biomarkers for the risk of HCC development would be of value.

During the period of chronic inflammation, intracellular reactive oxygen species (ROS) are increased and have a strong capability to induce oxidative DNA damage [20]. In this setting, the highly conserved phosphatidylinositol-3-related kinases ATM and ATR play critical roles in regulating the cell cycle checkpoints and DNA repair [21]. Of note, both of these kinases have a strong capability to increase the level of phosphorylated histone H2AX (*γ*-H2AX), which immediately traffics to the damaged sites of DNA. *γ*-H2AX plays a key role in the DNA repair process, because it recruits many other molecules involved in the DNA repair [17, 22]. Intriguingly, *γ*-H2AX is now regarded as a useful biomarker for assessing radio sensitivity of cancer cells after treatment [23], and more recently, it was reported that this unique molecule might be increased in preneoplastic lesions such as colon adenoma [24] and dysplastic lesions in the bronchial epithelium [25].

There have been few reports of *γ*-H2AX during hepatocarcinogenesis. Kim et al. [26] reported that *γ*-H2AX foci were significantly increased in HBV-related liver cirrhosis and HBV-related HCC compared with normal hepatocytes, but there have been no following reports and no such studies to investigate the clinical significance of *γ*-H2AX in individuals with a risk of HCC. In this study, we found that *γ*-H2AX was sequentially increased from normal to chronic hepatitis and liver cirrhosis. Importantly, dysplastic nodule showed a significantly high level of *γ*-H2AX LI, which was increased compared with HCC (74.1 ± 22.1% versus 56.2 ± 31.4%, *P* < 0.005). Together with our data suggesting that histological grades of HCC were inversely correlated with the level of LI, *γ*-H2AX might play a critical role in the development of HCC, especially during the early stages of carcinogenesis. Furthermore, the levels of LI increased in CC-coexisting tissues with liver cirrhosis than those without tumors (62.5 ± 24.7% versus 56.2 ± 31.4%, *P* < 0.005). Together, *γ*-H2AX may be a useful biomarker for predicting individuals with a high risk of HCC.

Until recently, there have been no useful biomarkers for predicting the potential of HCC development. Because *γ*-H2AX foci can be representatively detected using standard pathological techniques, this may be a promising and standard biomarker for HCC surveillance. To confirm the clinical usefulness of this DNA damage sensor, repeated liver biopsies and a long followup study of individuals with chronic liver diseases would be required.

## Figures and Tables

**Table 1:** Clinical characteristics of the patient groups of DN and HCC.

Clinical variables	DN (*n* = 19)	HCC (*n* = 58)	*P* value*
Mean age (year)	63 ± 8	62 ± 9	0.792
Gender			
Male	13	49	
female	6	9	0.117
HBs Ag			
+	2	7	
−	17	51	0.610
Anti-HCV			
+	12	35	
−	7	23	0.525

DN: dysplastic nodule; HCC: hepatocellular carcinoma.

*P value of independent Student's *t-*test for continuous data and Chi^2^ test for categorical data.

**Table 2:** Associations of *γ*-H2AX with clinicopathological features in HCC.

Clinicopathological variables	*γ*-H2AX immunoreactivity		
	Low (*n* = 18)	High (*n* = 40)	*P* value*
Age (year)			
<50	2	4	
≥50	16	36	0.613
Gender			
male	15	34	
female	3	6	0.576
Tumor size (cm)			
<3	5	11	
≥3	13	29	0.609
Intrahepatic metastasis			
−	14	30	
+	4	10	0.550
Venous invasion			
−	16	37	
+	2	3	0.497
Histological grade^†^			
I/II	10	35	
III/IV	8	5	0.011*

^†^ Histological grade was assessed according to the Edmondson-Steiner grade.

*P value of Chi^2^ test.
